# Obesity and male breast cancer: provocative parallels?

**DOI:** 10.1186/s12916-015-0380-x

**Published:** 2015-06-04

**Authors:** Matthew P. Humphries, V. Craig Jordan, Valerie Speirs

**Affiliations:** 1 grid.9909.90000000419368403Leeds Institute of Cancer and Pathology, University of Leeds, St James’s University Hospital, Leeds, LS9 7TF UK; 2 grid.240145.60000000122914776Department of Breast Medical Oncology and Molecular and Cellular Oncology, MD Anderson Cancer Center, Houston, TX 77030 USA

**Keywords:** Aromatase, Aromatase inhibitor, Body mass index, Estrogen, Incidence, Male breast cancer, Obesity, Risk factor, Selective estrogen receptor modulator, Tamoxifen

## Abstract

While rare compared to female breast cancer the incidence of male breast cancer (MBC) has increased in the last few decades. Without comprehensive epidemiological studies, the explanation for the increased incidence of MBC can only be speculated. Nevertheless, one of the most worrying global public health issues is the exponential rise in the number of overweight and obese people, especially in the developed world. Although obesity is not considered an established risk factor for MBC, studies have shown increased incidence among obese individuals. With this observation in mind, this article highlights the correlation between the increased incidence of MBC and the current trends in obesity as a growing problem in the 21^st^ century, including how this may impact treatment. With MBC becoming more prominent we put forward the notion that, not only is obesity a risk factor for MBC, but that increasing obesity trends are a contributing factor to its increased incidence.

## Background

To our knowledge, male breast cancer (MBC) was first reported in the medical literature in 1843 and described as the cause of death of five men in Paris from 1830 to 1840 [1]. Starting in the early 1800s, publications on MBC have steadily continued to increase from one or two each year to a peak of 68 publications in 2014. Data was obtained from PubMed using the search term “male breast cancer” in titles and abstracts from January 1st to December 31st, 2014 (search performed December 22nd, 2014).

According to the most recent estimates by The International Agency for Research on Cancer, a division of the World Health Organization, the global incidence of MBC stands at nearly 8,000 cases. For Europe, this equates to 3,750 cases of MBC [2]. In the US, the estimated number of cases in 2014 was expected to be 2,360, while the lifetime risk of men getting breast cancer is 1 in 1,000 [3]. These figures are significantly lower (<1 %) than the incidence of breast cancer in women, which represents 11.6 % of the global cancer incidence in 2014 [4], with approximately 232,000 and 425,000 women diagnosed with breast cancer annually in the US and Europe, respectively [5, 6]. Men typically present, on average, 5 years later than women, commonly in the seventh decade [7–9].

## Increasing incidence of male breast cancer (MBC)

**Fig. 1:**
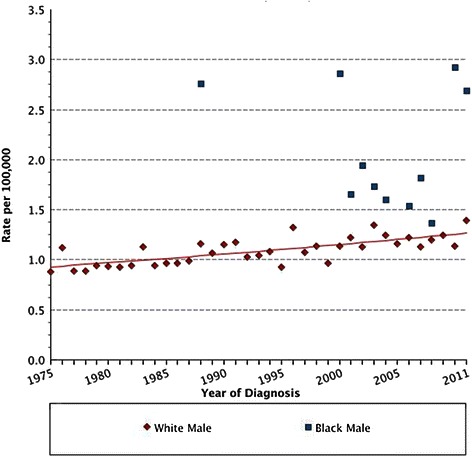
Age-adjusted increasing incidence rates in white men over a 36 year period (Diamonds), with the incidence rates in black men over the same time period overlaid (Squares). Data obtained and graphs generated from SEER using the Fast Stats Registry, National Cancer Institute

**Fig. 2:**
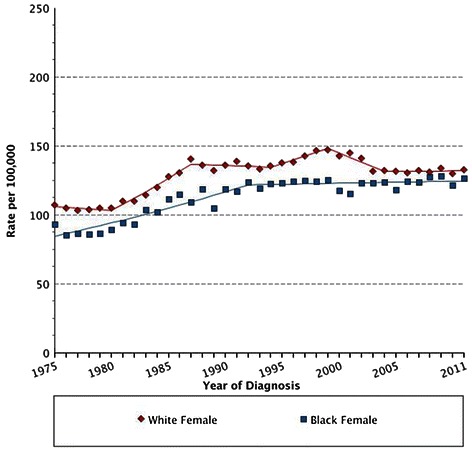
Age-adjusted increasing incidence rates in white women over a 36 year period (Diamonds), with the incidence rates of black women over the same time period overlaid (Squares). Data obtained and graphs generated from SEER using the Fast Stats Registry, National Cancer Institute

Studies suggest that MBC incidence is rising [10–14]. A large population-based study of 2,537 men with breast cancer, analysed from the National Cancer Institute’s Surveillance, Epidemiology and End Results (SEER) database, reported that over a 25 year period (1973–1998) the incidence of MBC increased significantly from 0.86 to 1.08 per 100,000 population in the US [15], with higher incidence in black than white men [16]. We have confirmed this in a more recent interrogation of the SEER dataset (Fig. 1) [17]. In terms of race, the rates of breast cancer are higher in black than white men while the reverse is true in female breast cancer (FBC) patients (Fig. 2). It should be noted from Fig. 2 that the incidence rates of FBC appear to plateau, which may be attributed to introduction of breast screening programs in the female population [18]. The higher rate of MBC observed in black men is at least partly due to more advanced disease presentation; studies have reported that a higher number of black MBC patients present with larger tumours of higher grade, lymph node metastases, and lacking in hormone receptor expression, compared to white men [19]. Such racial disparity may be associated with biological/genetic predispositions or socioeconomic factors such as access to health care [16, 20, 21], highlighted recently by work addressing disparities between black and white men with early stage MBC [22].

**Fig. 3:**
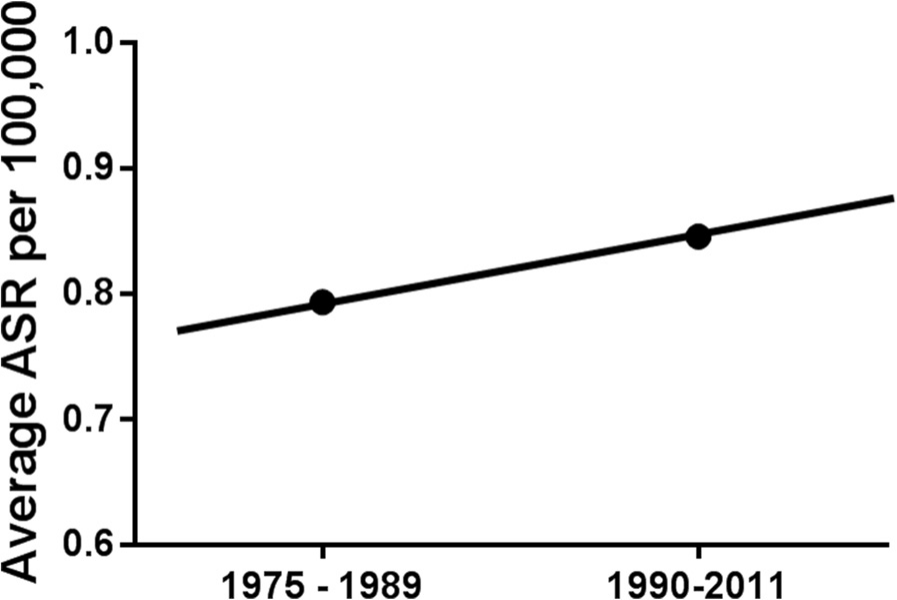
Average age-standardisation incidence rates (ASR) of male breast cancer in the UK per 100,000 in the 1970s and 80s vs 1990–2011. Average rate in the 1970s and 1980s was 0.79/100,000, while the average rate in 1990 to 2011 was 0.85/100,000. Figure generated from raw data taken from Cancer Research UK [21]

While at first glance data from the UK (Cancer Research UK) suggests no change in age-standardised incidence from 1975–2011 [23], when grouped into decades, there is a rise in the average age-standardised incidence of 0.79/100,000 population in the 1975–1980 to 0.85/100,000 from 1990–2011 (Fig. 3).

It is possible that perceived increases in MBC incidence may simply reflect increased disease detection. Men are generally more health conscious than in previous generations, perhaps aided by increased awareness of breast cancer in general, and public education in MBC specifically, for example, through the John W Nick Foundation [24]; part of their mission statement is “to educate the world about the risk of breast cancer in men”. Such campaigns may encourage men to present to a physician with breast-related symptoms, potentially increasing detection rates. Age, the single biggest risk factor for breast cancer, should also be considered; as an ageing population, increased MBC may well parallel increased longevity. However, increase in MBC is also reflected in age-standardised rates [15], which probably negates this argument.

## Obesity

**Fig. 4:**
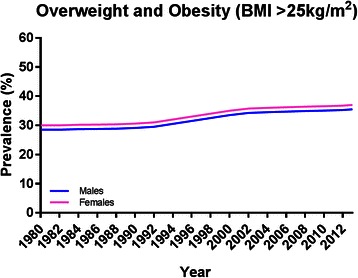
Increase in the global prevalence of obesity in males and females ≥20 years old from 1980–2013. Image adapted using data from [25]

One of the most worrying global public health issues is the exponential rise in the number of overweight and obese people, which in a comprehensive study of 1,769 reports from 188 countries showed a rise from 857 million in 1980 to 2.1 billion in 2013 [25] (Fig. 4); this has been described as “a global pandemic” [26, 27]. A key fact from these data is that, over this time period, more men than women were classified as overweight or obese in developed countries, whereas the opposite was true in developing countries [25]. The prevalence of overweight and obesity (overweight = body mass index (BMI, calculated as weight in kilograms divided by the square of height in meters) ≥25; obese = BMI ≥30) were highest in the World Health Organization regions of the Americas (62 % for overweight in both sexes, and 26 % for obesity); 65 % of the world’s population live in countries where overweight and obesity kills more people than underweight [28]. This trend shows no signs of reversing or even slowing as worldwide childhood obesity prevalence in 2010 was 6.7 %. Worryingly, in the United States, 20 % of children are categorised as obese [29].

## Obesity and cancer

It has long been known that obesity and the risk of cancer are linked. The International Agency for Research on Cancer and the World Cancer Research Fund reports show that common cancers in obese people are predominantly endometrial, esophageal adenocarcinoma, colorectal, postmenopausal breast, prostate, and renal [30, 31]. Recent data from the US provided evidence that the number of white men considered overweight or obese was higher than that of black men (BMI ≥25). However, when higher BMI was observed (BMI ≥30) the converse was true. Furthermore, there were 55 % more extremely obese black than white males (BMI ≥40) [32]. Interestingly, this increase in male obesity in developed countries seems to mirror an increase in incidence of MBC [25, 33, 34]. This is also reflected by comparing data in Figs. 1 and 4, where respective increases in the incidence of MBC and male obesity show parallel trends. In women, there is substantial and convincing evidence that weight gain at or around the menopause is a strong risk factor for breast cancer [35–39]. New evidence has emphasised this wherein obesity constituted an independent, adverse prognostic factor in node-positive FBC; this was particularly significant in estrogen receptor positive (ER+) postmenopausal women [40]. Furthermore, increasing incidence of FBC is seen in developing countries where Western lifestyles are increasingly adopted [41]. It should be noted that, while obesity is a risk for FBC, other known risk factors include changes in reproductive practices, duration of breast feeding, and administration of hormone replacement therapy [19]. As these do not apply to men, could the parallel rises of obesity and MBC incidence be linked? The case has been made that the increased incidence is simply a pseudo observation due to an increase in detection [42], which may well be true; however, without comprehensive epidemiological studies the rationale for the increased MBC incidence can only be speculated.

## Known risk factors for male breast cancer (MBC)

**Fig. 5:**
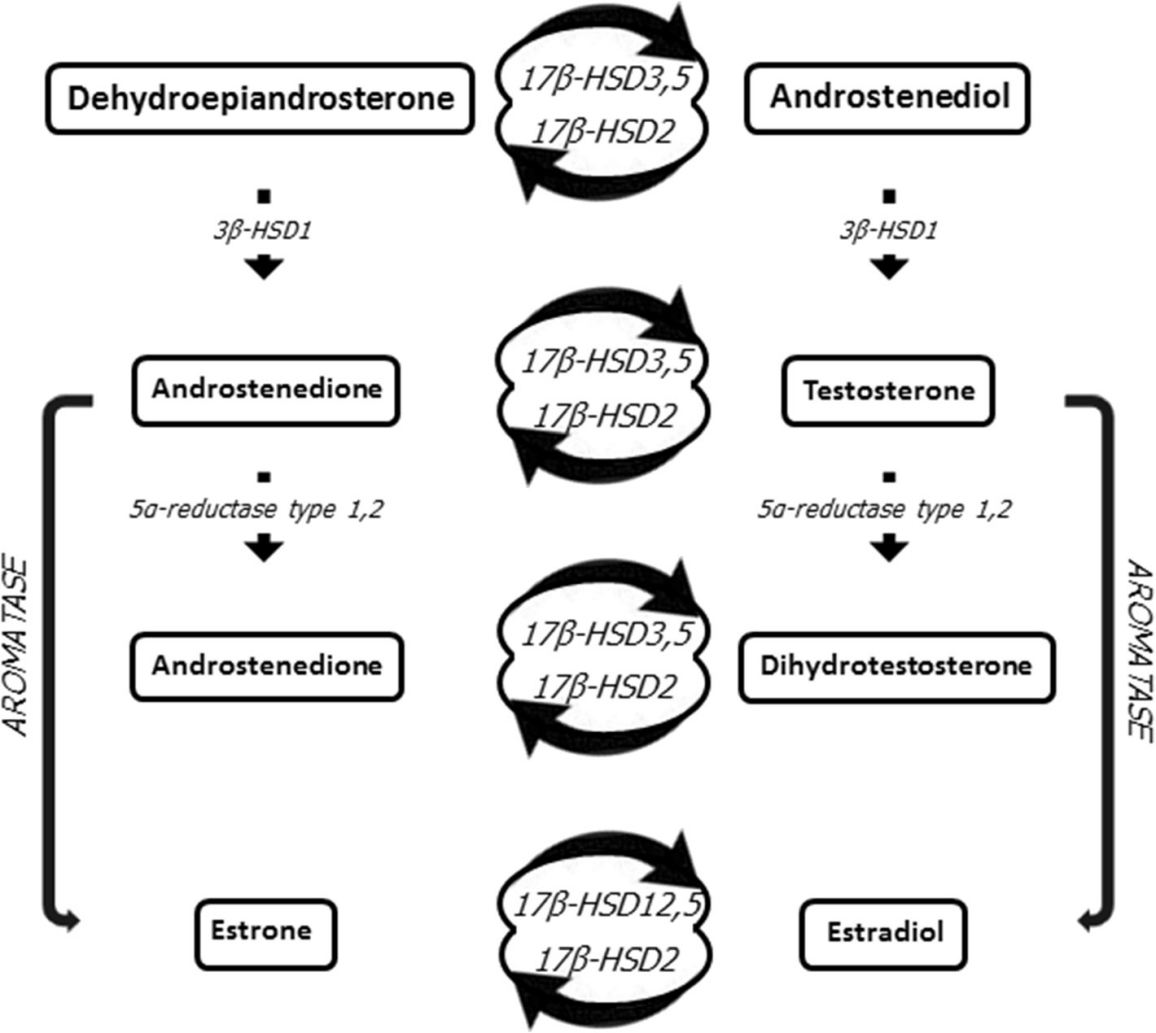
Pathways of steroid hormone metabolism in adipose tissue. Bold boxes highlight steroid hormones. Italics denote enzymes with arrows indicating direction of metabolism. Aromatase enzyme highlighted in bold italics. 3β-HSD1, 3β-hydroxysteroid dehydrogenase type 1; 17β-HSD2, 17β-hydroxysteroid dehydrogenase type 2; 17β-HSD3, 17β-hydroxysteroid dehydrogenase type 3; 17β-HSD5, 17β-hydroxysteroid dehydrogenase type 5; 17β-HSD12, 17β-hydroxysteroid dehydrogenase type 12. Image was compiled by the author by drawing on information from [56, 116, 117]

Risk factors associated with MBC include age, underlying genetics, including Klinefelter syndrome and BRCA2 mutations, radiation exposure, high estrogen levels, and liver cirrhosis as a result of excess alcohol consumption [7, 13–15, 33, 43–47]. However, a more recent publication has cast doubt on this latter association [48]. Gynecomastia is a relatively common benign enlargement of the male breast affecting an estimated 40–65 % of males [49], with some studies considering this a risk factor for MBC development [33] by as much as 10-fold [14]. Interestingly, gynecomastia is often associated with male obesity [50, 51]. Though not officially recognised as a risk factor for MBC, obesity has been highlighted by several studies as being significantly associated with MBC [14, 47, 52]. When one considers that adipose tissue contains aromatase, which converts testosterone to estrogen, increased levels of estrogen could produce an environment for cancer initiation in male breast tissue. Furthermore, conversion of testosterone to estradiol by aromatase in adipose tissue suppresses the release of luteinizing hormone, in turn leading to a reduction in testosterone production [53, 54]. As a result, estrogen levels are significantly higher in older men than post-menopausal women [55]. Adipose tissue is a recognised site for the production of steroid hormones. Aromatase is one of several enzymes found in adipose tissue and is involved in estrogen biosynthesis by converting testosterone to estrogen (Fig. 5). In addition to aromatase, other steroid-metabolising enzymes, including 17β-hydroxysteroid dehydrogenases, play a role in increasing local estradiol concentrations [56]. Notably, localization of both aromatase and 17β-hydroxysteroid dehydrogenase type 1 has been observed in MBC [57]. Adipose tissues are also recognised for their ability to secrete other factors, such as cytokines, which can enhance local estrogen biosynthesis that may exert local and/or systemic effects [58–64]. Recent data from the Male Breast Cancer Pooling Project supports the importance of estrogen in MBC etiology [65].

In the obese male, excess adipose tissue provides an environment conducive to increased estrogen production. Indeed, obese men produce as high as 2-fold more estrogen than men with average BMI [66] and males with very high BMI (≥35) show markedly decreased testosterone levels and increased concentrations of estradiol in blood plasma [15, 67–71]. Thus, weight increase coupled with the declining levels of hormones in the ageing male could provide a hormone environment which helps promote the genesis of MBC in obese men. In addition, a study by Brinton et al. [14], which included results from the Male Breast Cancer Pooling Project, provided evidence from over 2,400 patients from 10 cohort studies indicating that obesity was a positive risk factor for MBC; the authors observed a 30 % increased risk in MBC from obesity. This increased risk was in agreement with studies in post-menopausal FBC demonstrating a similar increased risk in cancer [72–74].

## Survival rates and treatment for male breast cancer (MBC)

MBC survival rates are generally assumed to be lower than in FBC, probably as a result of later diagnosis and the assumption that treatments developed through trials for FBC patients will perform equally well in men [75]. A study of over 13,000 men and almost 1.5 million women with breast cancer were reviewed over a 9 year period and showed 5 year survival rates of 74 % for men and 83 % for women [76]. The stage at diagnosis was a determining factor in survival rates for men compared to women. Latter stage diagnosis was more comparable, with 5 year survival in men at 16 % and women at 19 %. [76]. A European study including almost 500,000 women and over 2,500 men concluded that survival was worse in men than women until adjusting for age, stage and treatment, whereby the opposite was true [19]. This is in agreement with other smaller studies [8, 77, 34]. Additionally, time to diagnosis is negatively impacted by the presence of obesity [78–80]. This is due, but not limited to, difficulties in self-detection and possible embarrassment in seeking medical consultation due to excess weight. Further, obese patients are more likely to postpone clinical appointments for 3 months following first symptom occurrence [81]. Furthermore, obese patients undergo MRI and ultrasound less often when compared to those within a normal weight range [82]. All of the above may lead to more advanced disease at presentation which could impact survival.

The current standard of care for MBC patients is tamoxifen [83–87]. The case could be made that, when obese males present with MBC, aromatase inhibitors (AIs) should be advocated. Indeed, some studies have encouraged this for ER+ MBC patients in combination with tamoxifen [88]. However, this contradicts data from the ATAC (Arimidex, Tamoxifen, Alone or in Combination) trial, which compared the efficacy of AIs with tamoxifen, alone or in combination [89], where the combination treatment arm was closed after an initial analyses at 33 and 47 months of follow-up due to low efficacy [89, 90]. Granted, the ATAC trial was female-only but with growing evidence of biological differences between MBC and FBC [57, 91–95], the outcome may well be different in men. However, there are difficulties in accruing for a gender-specific trial, as highlighted by the withdrawal of a phase II study (SWOG-S0511) due to poor accrual of MBC patients (ClinicalTrials.gov. Trial record: NCT00217659). Nevertheless, analysis of 257 hormone-receptor-positive German MBC patients treated with tamoxifen (n = 207) or AIs (n = 50) showed the overall survival was significantly better after adjuvant treatment with tamoxifen compared to AI [96].

No clinical studies we are aware of have reported on the treatment of obese MBC patients with tamoxifen and AIs. Obesity in MBC patients should be considered in the context of treatment. Treatment with AIs alone have been shown to increase LH and FSH levels, which may be deleterious due to increased substrate for aromatisation in some MBC patients and further exacerbate the situation [97–100]. It has been hypothesised that introducing a gonadotropin-releasing hormone analogue with AIs, should tumour progression be seen, could counteract the activity of the hormonal feedback loop [88].

Additionally, functional single nucleotide polymorphisms (SNPs) exist in the aromatase gene that can improve how breast cancer patients respond to AIs. These alterations in the wild-type and variant sequences result in different DNA protein binding capabilities and displayed different transcriptional activity, resulting in atypical estrogen production [101]. Furthermore, there is early evidence that altered estrogen levels can be modified by changes in BMI in patients with these SNPs in aromatase [102]. These data highlight the need for further experimental studies focusing on the biological consequences of obesity and how this may impact on MBC treatment.

It is pertinent at this juncture to bring to light the connection between cholesterol and its oxysterol metabolite 27-hydroxycholesterol (27HC) in the context of obesity. 27HC is a recognised selective ER modulator (SERM) and was the first endogenous ligand demonstrated as having SERM activity [103]. The conversion of 27HC from the hydroxylation of cholesterol is achieved by the cytochrome p450 enzyme, CYP27A1 [104]. The ligand binding of 27HC to ER in ER+ breast cancer has been shown to induce cell proliferation [105, 106]. As MBC is overwhelmingly ER+ it follows logically that patients with higher 27HC provide a favourable growth environment for tumour development. Circulating levels of 27HC correlate with cholesterol levels; however, in the obese patient, cholesterol is often much higher and consequently an increase in 27HC is reported [107]. The consequence for the obese male is that increased 27HC levels, as a direct consequence of higher cholesterol, could contribute to the development of MBC via the mitogenic signalling of 27HC in the male breast.

## Conclusions

While the case for linking obesity to MBC is strong, a number of other contributing factors should be discussed. Changes in the Western diet over the past several decades may not only have shifted the average body composition towards a more obese/overweight state [108], but also changed the dietary composition towards a high intake of refined carbohydrates, added sugars, fats, and animal-source foods. Increased body awareness may result in earlier visits to GPs than in previous generations to investigate a problem, which will contribute to increased detection rates. Exposure to so-called environmental oestrogens via diet, household products, perfumes and deodorants are considered by some as risk factors for breast cancer development as collectively, prolonged exposure to low doses of these compounds could provide a significant estrogenic stimulus to target tissues [109–112]. However, there are no substantive data to support this and this would not be gender-specific.

Based on current evidence, we propose that the incidence trends of both MBC and obesity are inextricably linked and, as others have postulated [14, 47, 52], obesity should be considered as a risk factor for MBC in the same way this has recently been proposed for prostate cancer [113]. When considering treatment, a contextual perspective should be adopted with the atypical microenvironment, created and exacerbated by obesity, taken into account. Obesity is recognised as a preventable condition and sufficient weight loss has been shown to flip the ratio of hormones to normal physiological levels in obese men (BMI ≥30) [114, 115]. Peripheral aromatisation of androgens in adipose tissue of obese men could provide a hormonal milieu which encourages tumour development. While the risks of MBC from obesity have not been quantified, we propose that men at risk from MBC, e.g., those with BRCA2 mutations or suffering from Klinefelter syndrome, should be made aware of the relative risk between MBC and obesity to take pre-emptive measures to reduce this risk.

## Abbreviations

27HC: 27-hydroxycholesterol
AI: Aromatase inhibitors
BMI: Body mass index
ER: Estrogen receptor
FBC: Female breast cancer
MBC: Male breast cancer
SEER: Surveillance, Epidemiology, and End Results
SERM: Selective estrogen receptor modulator
SNP: Single nucleotide polymorphisms
